# Delftibactin-A, a Non-ribosomal Peptide With Broad Antimicrobial Activity

**DOI:** 10.3389/fmicb.2019.02377

**Published:** 2019-10-15

**Authors:** Noa Tejman-Yarden, Ari Robinson, Yaakov Davidov, Alexander Shulman, Alexander Varvak, Fernando Reyes, Galia Rahav, Israel Nissan

**Affiliations:** ^1^Public Health Regional Laboratory, Southern District, Ministry of Health (Israel), Beer Sheva, Israel; ^2^Laboratory Department, Ministry of Health, Jerusalem, Israel; ^3^Infectious Diseases Unit, Sheba Medical Center, Ramat Gan, Israel; ^4^The Mina and Everard Goodman Faculty of Life Sciences, Bar-Ilan University, Ramat Gan, Israel; ^5^Fundación MEDINA, Granada, Spain; ^6^Sackler Faculty of Medicine, Tel Aviv University, Tel Aviv, Israel; ^7^National Public Health Laboratory, Ministry of Health (Israel), Tel-Aviv, Israel

**Keywords:** delftibactin-A, antimicrobial, new antibiotic, *Delftia*, siderophore

## Abstract

The rapid emergence of drug resistant bacteria is occurring worldwide, outpacing the development of new antibiotics. It is known that some of the main sources of antibiotics are the bacteria themselves, many of which are secondary metabolites of Gram positive bacteria. Siderophores, which are secondary metabolites, function as natural chelators (e.g., iron). They are produced and secreted by many bacteria and have been experimented on as “carriers” of several types of antibiotics that pass the cell membrane of challenging Gram negative bacteria. Delftibactin A is a non-ribosomal peptide (NRP), which is known to detoxify gold in *Delftia* spp. and form gold nuggets, and is considered to be a siderophore. In this study we demonstrate that the supernatant from novel environmental isolates of *Delftia* spp. have antimicrobial activity. We characterized the active fraction and identified delftibactin A as a compound with antimicrobial activity. Delftibactin A exhibits potent antimicrobial activity against Gram positive multi drug resistant (MDR) bacteria like Methicillin-resistant *Staphylococcus aureus* (MRSA), and Vancomycin resistant *Enterococcus* (VRE), and also against the Gram negative pathogens *Acinetobacter baumannii* and *Klebsiella pneumoniae*. We discovered that the production of delftibactin A is greatly influenced by temperature. Furthermore, we have demonstrated the possibility of utilizing delftibactin A as a siderophore carrier of toxic metals such as gallium into Gram negative bacteria. These findings expose new opportunities of yet unexploited natural products such as delftibactin A, which have been known for other bacterial uses, as potent factors in the battle against MDR bacteria.

## Introduction

The fast and dramatic emergence of drug resistant bacteria is endangering the efficacy of antibiotics globally. The World Health Organization (WHO) have classified a number of bacteria as presenting urgent, serious, and concerning threats, many of which are already responsible for placing a substantial clinical and financial burden on the world health care systems, patients, and their families (World Health Organization [WHO], 2017).

During routine microbial testing of imported bottled mineral water, we notice the appearance of a large number of atypical small shiny colonies on mEndo Agar LES in some of the bottles. These peculiar colonies were later identified by 16S rRNA sequencing as belonging to the *Delftia* genus. Antibiogram generated using Vitek2 Compact revealed that these *Delftia* spp. isolates are multi drug resistance (MDR). We wondered if our *Delftia* isolates possess a resistance plasmid that can be transferred to other gut microbe by conjugation in people who drink the water. In order to test this hypothesis we used *Delftia* spp. as donor and *Escherichia coli* J5-3 as acceptor. Surprisingly, *Delftia* effectively killed the *E. coli*. In addition, we discovered the extraordinary phenomena of *Delftia* cells swarming toward target colonies (e.g., *E. coli, Staphylococcus aureus*), enclosing the target colony and lysing the cells (paper in preparation). This unique type of chemotaxis is termed predataxis. This serendipity led us to the discovery that *Delftia* spp. also secrete a library of compounds with antimicrobial activity- including delftibactin A which is the focus of the current study.

In this study we present a natural antimicrobial compound which we have purified from a new source, namely from the bacteria – *Delftia* spp. *Delftia* spp. are motile, Gram negative rods, which belong to the Betaproteobacteria lineage. Several *Delftia* species are mainly known for their biodegradation abilities of various toxic carbon compounds including phenols and aniline (Patil et al., 2006; Zheng et al., 2007; Zhang et al., 2008, 2010; Juarez-Jimenez et al., 2010, Juarez Jimenez et al., 2012) and as Plant Growth-Promoting Rhizobacteria (PGPR) – a group of rhizosphere bacteria that promote plant growth (Han et al., 2005; Morel et al., 2011; Ubalde et al., 2012; Hou et al., 2015; Guo et al., 2016; Perry et al., 2017; Cagide et al., 2018).

Bacteria belonging to the *Delftia* genus are rarely involved in human infections, mainly as opportunistic pathogens (Kawamura et al., 2011; Preiswerk et al., 2011; Mahmood et al., 2012; Ray and Lim, 2012; Shin et al., 2012; Ranc et al., 2018).

Delftibactin A is a non-ribosomal peptide (NRP). NRPs are secondary metabolites, which are biosynthesized by enzymes known as non-ribosomal peptide synthetases as opposed to the common path of peptide synthesis via the ribosome. Several NRPs are in therapeutic use as immunosuppressants, anticancer agents, and antibiotics for example vancomycin and daptomycin (Sussmuth and Mainz, 2017). The NRP-delftibactin A was recently identified as a probable siderophore which is capable of converting gold ions into molecular gold, thus detoxifying soluble gold as a mean of protecting the microbe (Johnston et al., 2013). Siderophores are natural compounds secreted by microorganisms including Gram positive and Gram negative bacteria as well as fungi. These molecules bind specific ions with great affinity, which is essential for bacterial survival. Siderophores can bind a variety of metal ions, including iron, copper, lead, gallium, etc.

Here, we demonstrate the antimicrobial activity of delftibactin A against multi-drug resistant (MDR) Gram positive pathogens like MRSA and VRE, as well as *Acinetobacter baumannii* which poses a world-wide threat from the Gram negative group of bacteria. We isolated and purified delftibactin A from novel environmental *Delftia* spp. isolates. Our data suggest that delftibactin A could be a new therapeutic candidate in the arsenal of compounds whose purpose is overcoming resistance, used both as a compound with antibacterial activity and as a carrier of toxic ions into the target bacteria.

## Materials and Methods

### Bacterial Strains and *Delftia* spp. Isolation

*Delftia* spp. isolate D-75 and D-2189 were initially isolated from bottled mineral water during routine examinations in the Ministry of Health Public Health Laboratory, Southern District, using mEndo Agar Les (HyLabs). These were identified as *Delftia* spp. based on 16S rRNA sequencing and later by whole genome sequencing (WGS, Supplementary Figure 7S). The Whole Genome Shotgun project has been deposited at DDBJ/ENA/GenBank under the accession for *Delftia* isolates D-2189 and D-75 FLCF00000000.2 and ERR1314968, respectively.

Clinical isolate strains of: *A. baumannii*, MRSA-USA300, VRE 19001, were provided by the Clinical Microbiology Laboratory at Sheba Medical Center.

*Escherichia* coli ATTC29299, *A. baumannii* _MB5973, MSSA ATTC29213, MRSA_MB5393, and *Klebsiella pneumoniae* ATTC700603 were used for MIC tests performed by Fundación MEDINA.

### Size Characterization of the Active Compounds

In order to elucidate the size of the molecules of the active fraction, D-75 and D-2189 were inoculated into 0.5 L Erlenmeyer flask with 100 mL Nutrient broth (NB, Difco catalog 234000) for 3 days at 30°C without shaking. Clear supernatants were collected as follows: the 3-day culture was centrifuged at 1500 g for 10 min at 4°C, and the clear supernatant (total supernatant) was filtered through a 0.45 μm membrane. The total supernatant was uploaded into a 3 kDa Amicon filter (Amicon Ultra-15, Ultracel-3K, catalog UFC900324) and centrifuged at 4,000 *g* for 40 min at 4°C. The activity of both the upper unfiltered fraction and the flow-through were examined for inhibitory activity against different target strains.

### Growth Conditions and Supernatant Collection

In order to simplify the purification of the active compound we optimized the cultivation of *Delftia* in defined minimal media. The isolates were cultured in Davis medium (Davis, BD catalog number 275610) supplemented with 0.5% glycerol (Sigma, catalog G7893-500ml) for various time periods at 30 or 37°C without shaking. Optical density was measured using either Amersham Bioscience Ultrospec 10 (600 nm) or TECAN GENios at 560 nm.

For delftibactin A production, several isolated colonies of *Delftia* clone D-2189 were inoculated into 0.5 L Erlenmeyer flasks with 100 mL of Davis medium supplemented with 0.5% glycerol and incubated for 8 days at 30°C in a water bath without shaking. At the end of the incubation period the culture was divided into two 50 mL tubes and the cells were separated from the supernatant by centrifugation at 1,500 *g* for 5 min. The clear supernatant was filtered using a 0.22 μM filter. The sterile supernatant was further purified by Amicon Ultra – 15 Centrifugal Filters (catalog number UFC 900324, 3,000 NMWL). 15 mL of sterile supernatant were uploaded to each filter and centrifuged at 4,000 *g* for 40 min at 4°C. The flow through was combined and lyophilized before HPLC chromatography as indicated below.

### Resistance to Proteinase K

Proteinase K (Sigma-Aldrich P6556) was added to clear supernatant of D-2189 and D-75 to a final concentration of 200 μg/mL in a total volume of 1 mL. Tubes with and without proteinase K were incubated for 6 h at 37°C. At the end of the incubation the proteinase K was removed from the sample by uploading the supernatant onto 3 kDa Amicon filters and cold centrifugation. The flow-through of the treatments and controls were used for the inhibition activity experiment.

### Resistance to RNAse A and DNAse I Assay

1 mL of D-75 supernatant was incubated with 10 μg/mL RNase A (Merck RNASEA-RO Roche) for 15 min at 60°C. A control supernatant was incubated as described without RNase A. At the end of the incubation period the RNase A was removed from the sample by uploading the supernatant onto 3 kDa Amicon filters and cold centrifugation. The flow-through was used for the inhibition activity experiment. The sensitivity of *Delftia* supernatant to DNase 1 was tested by addition of 25 μL DNase-1 (Qiagen kit catalog 79254) to 1 mL of D-75 supernatant and incubation for 10 min at room temperature. A control tube was incubated as described. The enzyme was removed from the media using additional centrifugation with the 3-kDa Amicon membranes. The OD_600_ nm of USA300 cultures was measured after 6 h of aerobic incubation with the RNAse A/DNAse 1 treated supernatant at 37°C, with shaking (150 RPM).

### HPLC Analysis and Comparison of Supernatants From 30 and 37°C Cultures

Clear supernatant from D-75 and D-2189 were collected as described above. The 3 kDa Amicon Ultra – 15 Centrifugal filtered supernatants were lyophilized and reconstituted in double-distilled water (DDW) and were analyzed using a Merck Hitachi Lachrom HPLC system controlled by EZChrom Elite software. For analytical separation we used a Kinetex^®^ 5 μm EVO C18 100 Å, LC Column 150 × 4.6 mm and an injection volume of 5–10 μL. For production of larger amounts we used C-18 semi-preparative column (Kinetex^®^ 5 μm EVO C18 100 Å, AXIA Packed LC Column 150 × 21.2 mm) at a flow rate of 9.9 ml/min and an injection volume of 100–500 μL. In both cases, the RP-HPLC method employed linear gradient elution using two mobile phase buffers: Buffer A, 0.1%TFA in acetonitrile, and Buffer B, 0.1%TFA in water. The gradient was as follows: 0–3 min, 100% Buffer B, 3–17 min, 100–85% Buffer B, 17–18 min, 85 to 5% buffer B, after which the column was re-equilibrated to initial conditions over a period of 7 min. The UV absorbance signal was collected by a Hitachi L- 7455 diode array detector in the range 200–400 nm. The chromatograms at wavelengths of 220 and 280 nm were used to detect the peaks. Fractions were collected using a Foxy R1 fraction collector (Teledyne Isco) and dried for further characterization.

### LC/HRMS

Samples were dissolved in 200 μL of water and analyzed by LC/HRMS using an Agilent 1200 Rapid Resolution HPLC interfaced to a Bruker maXis mass spectrometer. The volume of sample injected was 4 μL. A Waters Atlantis T3 column (4.6 × 100 mm, 5 μm particle size) was used for the separation. Two solvents were used as mobile phase: solvent A water:AcN 90:10, solvent B water:AcN 10:90, both with 13 mM ammonium formate and 0.01% TFA. The gradient started with a 100% of A, went to 100% of B in 11 min, was held at thata composition for 5 min and went back to 100% A in o.1 min and reequilibrated at that percentage for 3.9 min. A flow of 0.5 mL/min was used.

The mass spectrometer was operated in positive ESI mode. The instrumental parameters were: 4kV capillary voltage, drying gas flow of 11 L/min at 200°C, nebulizer pressure at 2.8 bars. TFA-Na cluster ions were used for mass calibration of the instrument prior to samples injection. Each sample run was recalibrated by infusion with the same TFA-Na calibrant before the chromatographic front. Each chromatographic run was processed using the Bruker algorithm for components extraction, and the most intense peaks of each run, either by UV or positive Total Ion Current (TIC) were considered for interpretation of exact mass and molecular formula by studying the extracted mass components.

### NMR

NMR spectra were acquired at 24°C in CD_3_OD using a Bruker Avance III 500 MHz spectrometer equipped with a 1.7 mm micro-cryoprobe. Solvent residual signals (3.31 ppm for ^1^H and 49.15 ppm for ^13^C were used as reference).

### Minimal Inhibitory Concentration (MIC) Determination

Delftibactin A was dissolved in 100% dimethylsulfoxide (DMSO) to a stock solution of 4 mg/mL. The compound was further diluted in DMSO and tested in triplicate at final concentrations between 64 and 0.125 μg/mL. A volume of 90 μL of the appropriate diluted inoculum (about 5 × 10^5^ CFU mL^–1^) of the tested pathogen in cation-adjusted Mueller-Hinton Broth CAMHB (BBL Mueller Hinton II Broth, catalog 212322), was mixed with 8.4 μL of CAMHB and 1.6 μL of the delftibactin A dilutions (total volume 100 μL per well). Delftibactin A inhibitory activity was tested against MRSA (methicillin-resistant *S. aureus*), MSSA (methicillin-sensitive *S. aureus*), *A. baumannii*, *Pseudomonas aeruginosa*, *E. coli*, and *K. pneumoniae*. Rifampicin (25–3.125 μg/mL), aztreonam (2–0.25 μg/mlL), gentamicin (32–4 μg/mL), vancomycin (32–4 μg/mL) and penicillin G (0.312–0.039 μg/mL) were used as positive controls for *A. baumannii*, *E. coli*, *P. aeruginosa*, *K. pneumoniae*, MRSA and MSSA, respectively. Amphotericin B (16 μg/mL) was used as negative control for all the cases. Experiments were conducted at 37°C. Absorbance at OD_612_nm was measured with Envision reader (Perkin Elmer) at zero time (T_0_) and at final time (T_f_). Percentage of growth inhibition was calculated according to Audoin et al. (2013). The MIC is defined as the lowest concentration of compound that inhibited ≥90% (MIC90) or ≥50% (MIC50) of the growth of a microorganism after 16 h of incubation at 36 ± 1°C.

### Assessment of Supernatant Activity

Few colonies of the target strain were picked from a fresh plate into Nutrient Broth (NB) and the OD_600_ was adjusted to 0.1. The bacterial inoculums were further diluted 1:100 in NB. 330 μL of the bacterial inoculum were mixed with 660 μL of the examined supernatant, and incubated at 37°C in a water bath, with a gentle shake for 6 h. After incubation, OD_600_ was determined and recorded.

### Co-incubation of Clarithromycin and Rifampicin With Delftibactin A

Clarithromycin (Sigma-Aldrich C9742-100MG) was dissolved in acetone to a stock solution of 25 mg/mL. The clarithromycin was further diluted in NB to a final concentration of 0.5 μg/mL or below. The activity of clarithromycin was tested alone and in combination with delftibactin A at final concentrations of 10 μg/mL. Rifampicin (Sigma-Aldrich, catalog R3501-250MG) stock was prepared in DMSO (100 mg/mL) and further diluted in NB to a final concentration of 0.5, 0.25, 0.125 μg/mL and tested alone or in combination with an identical concentration of delftibactin A.

### Other Reagents

Gallium (III) nitrate hydrate (Sigma-Aldrich catalog 289892-5G), synthetic delftibactin A peptide ASDTGT[Orn]SR[Orn]- amide (H-Ala-Ser-Asp-Thr-Gly-Thr-Orn-Ser-Arg-Orn-NH_2_, Sigma-Aldrich, Israel), Iron (II) chloride (Sigma-Aldrich, catalog 157740-100G).

### Co-incubation of Gallium/Iron With Delftibactin A

Gallium/iron and delftibactin A were either added separately, simultaneously or pre-incubated together for 30 min at room temperature prior to addition into the test tube.

### Delftibactin A Cytotoxicity Assay

The assay was performed essentially as described by Cautain et al. (2015). Briefly, Hep G2 (ATCC^®^ HB-8065^TM^, Epithelial. Hepatocellular carcinoma) and THLE-2 (ATCC^®^ CRL-2706^TM^, epithelial cell line, human liver/left lobe) cell lines were seeded at a concentration of 2 × 10^4^ cells/well (96 well plate) in 200 μL culture medium and incubated at 37°C in 5% CO_2_. After 24 h, the medium was replaced with a final volume of 200 and 1 μL solution of the tested compounds (dilution 1/200) and controls were added to the plates. 8 mM methyl methanesulfonate (MMS), was used as positive control and DMSO 0.5% was used as negative control. As standard (internal control) a Doxorubicin curve was used. DMSO was tested in a preliminary experiment with these cell lines, and no effects were observed with 1% DMSO. After addition of delftibactin A and controls, plates were incubated for 24/72 h. MTT (3-(4,5-Dimethylthiazol-2-yl)-2,5-diphenyltetrazolium bromide) solution was prepared at 5 mg/mL in PBS 1X and then diluted 1:10–0.5 mg/mL with MEM without phenol red. The sample solution in wells was flicked off and 100 μL of MTT dye was added to each well. The plates were gently shaken and incubated for 3 h. The supernatant was removed and 100 μL of DMSO 100% was added. Absorbance was measured using a multireader Victor^TM^ at a wavelength of 570 nm.

## Results

### Secretion of *Delftia* Strain 2189 Antimicrobial Compounds Is Temperature Dependent

*Delftia* strain 2189 (D-2189) was incubated for 3 days at 30 or at 37°C in NB. The 0.45 micron filtered supernatant was tested for its inhibitory effect against MRSA USA300 and XDR *A. baumannii* (Supplementary Figure 1S). The entire inhibitory tests were performed at 37°C. MRSA and *A. baumanni* exposed to the 30°C supernatant were significantly inhibited compared to treatments with supernatant of D-2189 grown at 37°C. These results demonstrate that there are antibacterial compounds produced by D-2189 whose secretion is temperature regulated. Supernatants derived from 37°C cultures of D-2189 or D-75 serve as negative controls for antimicrobial activity (depleted media).

### Size and Biochemical Characterization of the Active Supernatant

*Delftia* strain 75 (D-75) supernatant was fractionated using 10 kDa (data not shown) and 3 kDa Amicon filters (Supplementary Figure 2S). The different fractions of the supernatant (flow-through supernatant and the upper fraction which did not pass the filters), were tested for MRSA USA300 inhibition. Most of the activity was present in the flow-through fraction containing molecules smaller than 3 kDa. Interestingly, this activity is significantly higher than that of the total supernatant, which may indicate higher specific activity by size purification. The activity of the smaller than 3 kDa fraction was confirmed against *A. baumannii* (Supplementary Figure 3S). Although most of the activity is in the fraction smaller than 3 kDa, some activity was also observed in the fraction larger than 3 kDa. In order to gain insight into the chemical nature of the active compounds, various treatments were applied to the active fractions, namely by exposing these fractions to nucleases, heat and proteinase K (Supplementary Figure 3S). The stability of the active supernatant was challenged using RNase and DNase (Supplementary Figure 3S). RNase A or DNase-1 do not affect the antimicrobial activity. Furthermore, no reduction in antimicrobial activity of the supernatants after incubation at 85°C for 30 min was detected (data not shown). In addition, proteinase K did not affect the compound activity either (Supplementary Figure 3S). Taken together, these results indicate that the active compound in the supernatant (smaller than 3 kDa) is not a nucleic acid nor a proteinase K sensitive protein.

**FIGURE 1:**
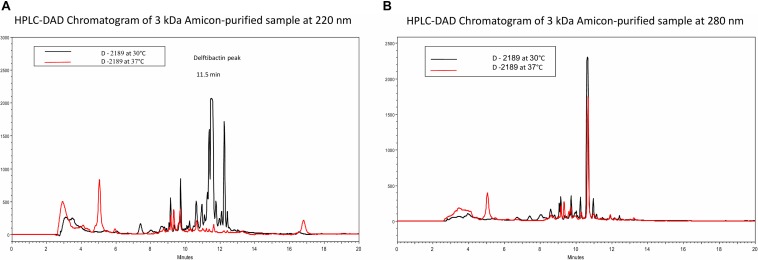
HPLC chromatograms of *Delftia* strain D-2189 supernatants grown in Davis minimal medium with 0.5% glycerol after 7 days incubation at 30°C (black line) and 37°C (red line). Significant differences are apparent at 220 nm **(A)**, with dominant peaks at 11.5 and 12.3 min in the active supernatant (30°C). In contrast, chromatograms of the same samples at 280 nm were very similar **(B)**.

Based on the difference in antimicrobial activity of supernatants of D-2189 and D-75 at different incubation temperatures, we sought to develop an analytical HPLC method for identifying the active compounds through observation of the differential peaks produced at 30 (and not at 37°C). In order to do so we optimized *Delftia* growth conditions in Davis minimal medium with various glycerol/phthalic acid concentrations (not shown). Davis minimal medium containing 0.5% glycerol (V/V) as the carbon source was found to be the best combination. Comparison between the chromatograms of D-2189 supernatant incubated at the different temperatures clearly indicate the existence of significant differences in the type and the amount of compounds secreted as seen by UV detection at 220 nm (Figure 1A). The most significant differential peaks appear at retention times 11.5 and 12.3 min at 220 nm. Importantly, at 280 nm the chromatograms of supernatants obtained at both temperatures, look very similar (Figure 1B). This result was used as an internal control for the separation process of the active compounds.

**FIGURE 2:**
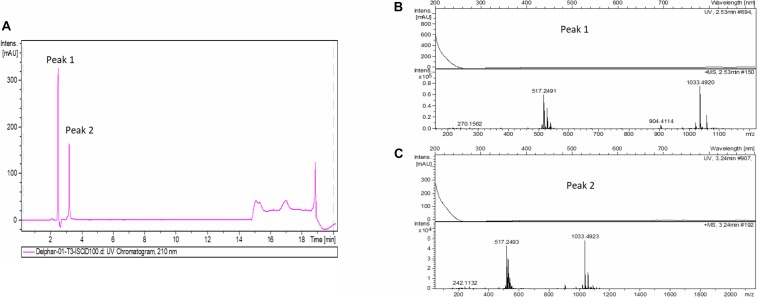
Identification of delftibactin A in the purified active sample: **(A)** Chromatogram shows the trace of UV absorbance at 210 nm for the purified active sample – fraction 11.5 min (UV chromatogram at 210 nm). **(B,C)** HRMS spectra of peaks 1 and 2 are shown.

**FIGURE 3:**
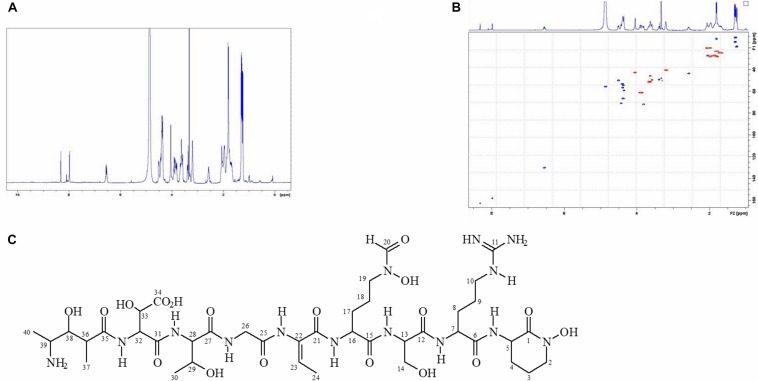
^1^H-NMR and HSQC spectra of delftibactin A **(A,B)** and structure of delftibactin A **(C)**.

In order to accumulate larger amounts of material we optimized a semi preparative HPLC method for the purification and identification of the active compound. Various HPLC-eluted fractions of D-2189 and D-75 were collected using a fraction collector every 0.5 min, lyophilized and re-suspended in NB for testing the inhibitory activity against MRSA USA300 (Supplementary Figure 4S). Prominent inhibitory activity was detected in fractions eluting at 11–12.5 min with maximal activity at 11.5 min. The final identification of the active compound was performed using LC/HRMS and NMR (Figures 2,3 and Supplementary Figures 10S, 11S). The chromatographic behavior of the compound under the LC/HRMS conditions tested exhibited two peaks (Figure 2A) containing protonated adducts at m/z 1033.4920 and 1033.4923 (Figure 2B), in accordance with a molecular formula of C_40_H_68_N_14_O_18_. A search of this molecular formula in PubChem retrieved a compound named delftibactin A that matched all the analytical data acquired (Figure 3C). The NMR spectra of the sample (acquired in CD_3_OD) showed the presence of a unique molecule, and were in agreement with those reported for delftibactin A in D_2_O (Johnston et al., 2013; Figure 3 and Supplementary Figures 10S, 11S). Two-dimensional COSY, HSQC and HMBC spectra helped to corroborate the identity of the amino acids forming the peptide whereas key HMBC inter-residue correlations confirmed the amino acid sequence. The ^1^H and ^13^C NMR spectra showed two different sets of signals for the protons and carbons of the *N*^δ^ -hydroxy-*N*^δ^ -formylornithine residue that likely correspond to the *cis* and *trans* rotamers of the formamide functional group (Supplementary Figure 11S). These two species are in dynamic equilibrium and interconvert every few tens of seconds. This is slow in the NMR time-scale, so two sets of signals are observed, but is fast as far as biological activity. Interestingly, according to the model presented by Johnston et al., 2013, the delftibactin A binding site for gallium ion is produced by the dominant *cis* isomer of formamide. The purity of delftibactin A fraction was found to be 93.8% (Supplementary Figure 12S).

### Antimicrobial Activity of Delftibactin A

**FIGURE 4:**
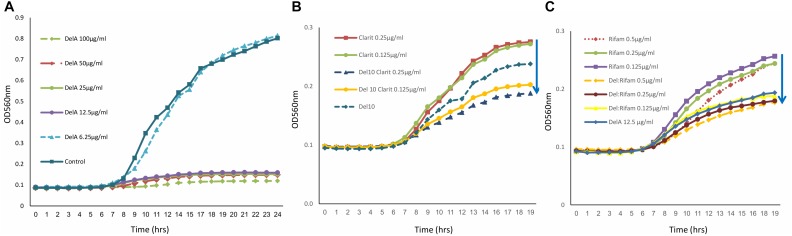
Graph illustrating that delftibactin A inhibits Vancomycin Resistant Enterococci (VRE) alone and in combination with other antibiotics. **(A)** Serial delftibactin A dilutions from 6.25 μg/mL up to 100 μg/mL were tested against VRE 19001. Total growth inhibition can be detected at 12.5 μg/mL. **(B)** Combination of delftibactin A (at constant concentration of 10 μg/mL) with various concentration of clarithromycin enhance clarithromycin activity. **(C)** The combination of delftibactin A and rifampicin at identical concentration of 0.5, 0.25, 0.125 μg/mL significantly enhance rifampicin activity.

The antimicrobial activity of purified delftibactin A was tested against several Gram positive and Gram negative bacterial targets. Delftibactin A inhibited the growth of MRSA (MB5393) and MSSA (ATCC-29213) strains with a MIC_50_ of 8 μg/mL, and also caused a total growth inhibition of VRE at 12.5 μg/mL (Figure 4A). Delftibactin A also exhibited inhibitory effect against challenging Gram negative bacteria *A. baumanni* (MB5973) and *K. pneumoniae* (ATCC-700603) with a MIC_50_ of 16 μg/mL. Lower potency was obtained against *P. aeruginosa* with an MIC_50_ of 32–64 μg/mL, and no significant inhibition of *E. coli* was observed at the highest concentration tested. MICs of control antibiotics appear at the Supplementary Figure 9S.

We continued to check the possibility of improving the potency of delftibactin A against the MDR Gram positive bacteria by combining it with other known antibiotics, the protein synthesis inhibitor clarithromycin belonging to the macrolide drug class (Piscitelli et al., 1992) and the DNA dependent RNA polymerase inhibitor polyketide rifampicin (Calvori et al., 1965). Both clarithromycin and rifampicin are effective against a range of Gram positive bacteria yet have a distinct mechanism of action. As can be seen, the combination of clarithromycin with delftibactin A (10 μg/mL) has an additive inhibitory effect against VRE in a dose dependent manner (Figure 4B). Remarkably, the combination with rifampicin looks very promising since a measurable additive inhibitory effect was achieved at very low concentration of both drugs (Figure 4C).

In order to simplify the production of delftibactin A we examined the efficiency of synthetic peptide with the backbone amino acid sequence of delftibactin A: H-Ala-Ser-Asp-Thr-Gly-Thr-Orn-Ser-Arg-Orn-NH_2__._ The activity of this synthetic analog of delftibactin A was tested against MRSA USA300 and *A. baumannii*. No inhibitory effect was detected up to the concentration of 1 mg/mL (Supplementary Figure 5S). We speculate that the reason why this synthetic analog of delftibactin A has no antimicrobial activity is because it lacks several modifications which are present in the natural compound.

**FIGURE 5:**
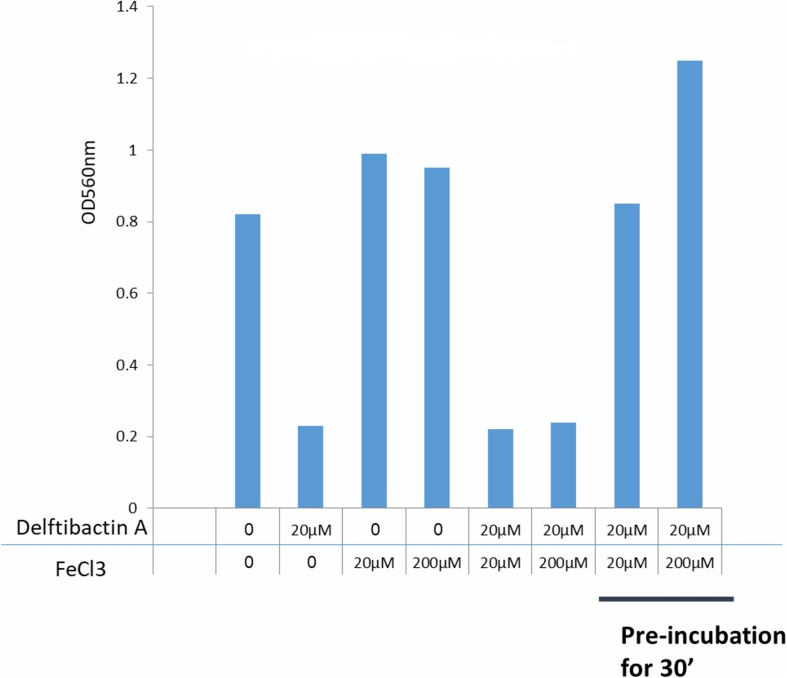
Effect of iron on the antimicrobial activity of delftibactin A tested on *Staphylococcus aureus* after overnight incubation. Adding iron to the media increases bacterial proliferation. Adding 10 fold higher molar concentrations of iron than delftibactin A, does not affect delftibactin’s inhibitory effect. However, when pre-incubated together, iron inhibits delftibactin’s antimicrobial activity.

It is known that delftibactins bind with high affinity to metals such as gold or gallium (Johnston et al., 2013; Wyatt, 2013). Furthermore, the production of delftibactin A is reversely proportional to the iron level (Wyatt, 2013). We therefore sought to determine the effect of iron on the antimicrobial activity of delftibactin A. As seen in Figure 5, addition of iron further induces *S. aureus* proliferation, nevertheless, supplementation of iron at the same molar ratio as delftibactin A or even ten-fold higher, does not interfere with the antimicrobial effect of delftibactin A. On the other hand, preincubation of iron and delftibactin A at equal and ten-fold molar ratios eliminates its antimicrobial effect. Similar results were obtained using the iron-mimetic gallium instead of iron. Pre incubation with gallium completely eliminates the antimicrobial activity of delftibactin A (Supplementary Figure 13S). These findings suggest that delftibactin A binds quickly to its target bacteria even when there is excess of iron in the culture medium.

**FIGURE 6:**
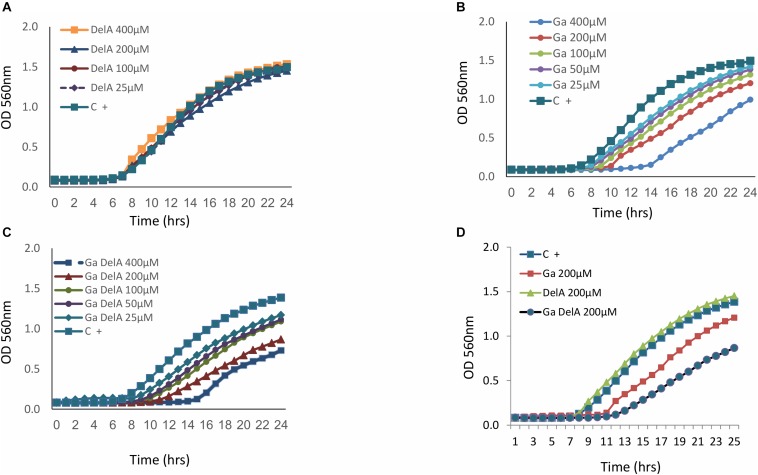
The inhibitory effect of delftibactin A with and without gallium on *Delftia* strain D-2189. Delftibactin A has no inhibitory activity against D-2189 **(A)**. Dose dependent inhibition of gallium against D-2189 **(B)**. Addition of isomolar concentration of gallium and delftibacin A induces significant inhibition of *Delftia* strain D-2189 in a dose dependent manner **(C)**. **(D)** Illustrates the combined effect of delftibactin A- gallium, by displaying the activities of: 200 μM delfibactin A only, gallium only and isomolar mixture of delftibactin A-gallium in the same graph.

As delftibactin A can “carry” iron, we tested the possibility of substituting it with gallium, which shares chemical properties with iron but has disruptive effects in bacteria (Antunes et al., 2012). Since delftibactin A also serves as a siderophore (Johnston et al., 2013; Wyatt, 2013) we assume that *Delftia* most probably express transporters for this molecule. Indeed, a probable delftibactin transporter was identified immediately downstream of the delftibactin biosynthesis cluster (Daci_4752 TonB-dependent siderophore receptor, Supplementary Figure 8S). Having gallium naturally conjugated to delftibactin A can act as a “Trojan horse” carrying a toxic compound into bacteria which possess the transporters for delftibactin A. In order to test this hypothesis, we used strain D-2189 as the model bacteria, since it produces delftibactin A and therefore most probably also expresses the transporters. This strategy resulted in an additional antimicrobial effect (Figure 6). Figure 6A demonstrates that delftibactin A alone does not inhibit *Delftia*, and gallium alone inhibits *Delftia* moderately (Figure 6B), whereas the combination of gallium with delftibactin A induces significant inhibition of *Delftia* growth (Figures 6C,D).

**FIGURE 7:**
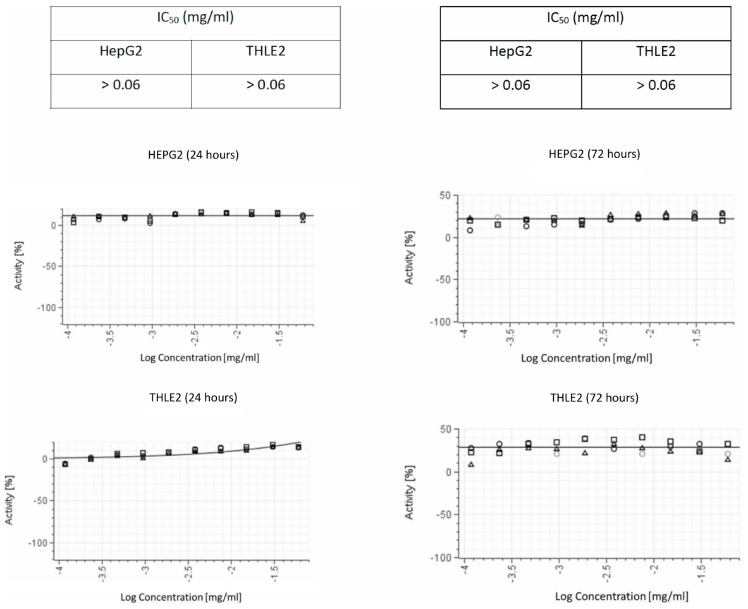
Cytotoxic activity in both THLE2 and HepG2 cell lines illustrating the results of viability assays on liver cell lines. The graphs on the left summarize the results of 24 hours exposure of the cells to purified delftibactin A, and on the right 72 hours exposure.

We ruled out non-specific toxic activity of delftibactin A alone using two different human cell lines (Figure 7). No inhibitory effect was detected after 72 h incubation with a concentration up to 0.1 mg/mL.

## Discussion

In this study we describe delftibactin A as a novel antimicrobial compound that is able to inhibit some of the major clinical MDR bacteria. We have isolated and purified delftibactin A from environmental isolates of *Delftia* spp. *Delftia* spp. secrete antimicrobial compounds to the supernatant in a temperature dependent manner; this active supernatant is stable to heating, nucleases and proteinase K treatments. Following several steps of fractionation, we found that the main component of the potent active fraction in the supernatant is delftibactin A. In addition, we identified yet other fractions in the supernatant which exhibited antimicrobial activity- meaning that *Delftia* spp. are capable of secreting other, yet to be determined, antimicrobial compounds to their surroundings. Delftibactin A is potent against a broad range of multi-drug resistant pathogens of both Gram positive and Gram negative clinical isolates including MRSA, MSSA, VRE and *A. baumannii*. Furthermore, combining delftibactin A with other antibiotics with different mechanisms of action significantly increases its antimicrobial effect. The combinations of delftibactin A with known antibiotics could on one hand, reduce the drug doses, thus reducing side effects caused by the different antibiotics. Furthermore, the combination of the two agents may reduce the probability for resistance buildup. We could not detect delftibactin A toxicity in various human cell lines. *Delftia* spp. are not hemolytic on blood agar. Nevertheless, the safety of delftibactin A treatment should be tested also in an animal model. As delftibactin A is stable to heat and various enzymatic treatments, we speculate that this impressive stability may also contribute to its stability *in vivo*.

Delftibactin A- has been lately reported for its ability to detoxify gold ions – thus protecting *Delftia* which produce and secrete it (Johnston et al., 2013). Detoxification of toxic gold ions by delftibactin A is an advantage only in very unique and rare niches. Interestingly, the non-ribosomal peptide synthetase gene *delG* (Daci_4754 of *Delftia acidovorans* DSM no. 39) a major gene in the delftibactin biosynthesis pathway (Johnston et al., 2013), was found in various beta proteobacteria, including the challenging pathogens *Burkholderia cepacia* complex (BCC), *Burkholderia pseudomallei* (Supplementary Figure 6S) and Variovorax (Hong et al., 2017). The large (6,176 amino acids) non-ribosomal peptide synthetase gene *delH* (Daci_4753) which is part of the delftibactin synthesis locus [Daci_4753-4759, *del* cluster (Johnston et al., 2013)] is also present in various human and plant pathogens (not shown). Plausibly, the production and secretion of delftibactins by these beta proteobacteria improves their fitness and competition capabilities in the various environmental niches.

The concept of using siderophores as carriers of antibiotics into Gram negative bacteria has been described before (Gorska et al., 2014; Tillotson, 2016). In this study we have shown that delftibactin A is not toxic to the bacteria that produce it, i.e., *Delftia* spp. As a siderophore, *Delftia* spp. probably expresses a delftibactin transporter. We tested this hypothesis by addition of the iron-mimetic gallium alone and in combination with delftibactin A. Gallium alone is toxic and moderately inhibits *Delftia* growth to some extent, but in combination with delftibactin A, the inhibitory effect was significantly enhanced. These results serve as the first indication for the presence of delftibactin transporter that is able to transport the delfibactin A-gallium complex into the bacterial cells. Therefore, another application for this unique NRP is using it as a “Trojan horse” that may be is use against challenging pathogens like BCC. Johnston et al. (2013) described downstream genes (from the *del* locus) that probably associate with metallophores that bind iron (siderophores) and, specifically, genes for their reception and regulation. We suggest that *Daci_4752* (TonB-dependent siderophore receptor) encodes for the delftibactin A transporter (Supplementary Figure 8S). TonB-dependent siderophore receptor are common in many Gram negative bacteria like *E. coli*, *Acinetobacter*, and *Pseudomonas* (PanDaTox collection at the Weizmann Institute of Science)^1^. We wonder if some of the bacteria possessing TonB-dependent siderophore receptors are able to bind and translocate delftibactin A-iron complex (xenosiderophore). The phenomena of xenosiderophore is well established for various Gram positive and Gram negative bacteria. In support of this hypothesis delftibactin A enhance the inhibitory activity of gallium against *A. baumanii* (not presented). A combination of delftibactin A or delftibactin family siderophore with various toxic metals like gallium may be used to treat these (above mentioned) highly resistant and hard to cure pathogens. This more simple and natural approach can bypass the need for complex conjugation phase of antibiotics to the siderophore (e.g., beta lactams, Chairatana et al., 2015).

Further testing of delftibactin A activity against a larger range of pathogens and elucidation of the mechanism behind its inhibitory abilities, should be done in the near future. Taking advantage of the different properties of this antimicrobial peptide of Gram negative origin, it could provide new tools in approaching multi drug resistant pathogens. Johnston et al demonstrated that *D. acidovorans* also produces delftibactin B (Johnston et al., 2013). We also identified low amounts of this compound as well as other delftibactin like molecules in the *Delftia* supernatants. It will be of great value to purify other members of the delftibactin family and to test their antimicrobial activity.

Our NMR data show that the formamide functional group of delftibactin A can exist in *cis* (dominant) or *trans* (minor) conformations (Supplementary Figure 11S). These two forms are in dynamic equilibrium and interconvert. According to the metal binding model of Johnston et al., 2013, only formamide group in its *cis* conformation participates in gallium binding. We question whether these two conformations of delftibactin A serve different biological roles.

We found that co-administration of iron and delftibactin A does not abolish the antimicrobial effect of delftibactin A whereas pre-incubation for 30 min completely abolishes it. Similar results were obtained using gallium. This indicates that the association of delftibactin A with the bacterial cells is very fast and stable, since even a ten-fold higher iron concentration does not interfere with this activity. On the other hand, since pre-incubation with iron or gallium abolished delftibactin A activity, we assume that metal binding induces a conformational change that prevents the association of the complex with the target pathogen but allows binding to delftibactin A transported and translocation into *Delftia* spp. The various conformations that delftibactin A can acquire may serve as a molecular switch which controls its activity.

In summary, we have expanded the knowledge about delftibactin A functions. Delftibactin A is a multifunctional NRP that is used by *Delftia* spp. as a siderophore, antimicrobial compound and for detoxification of gold ions. Our results also expand the knowledge about the activity of delftibactins in various beta proteobacteria that produce them and specifically about the Plant Growth-Promoting Rhizobacteria (PGPR) activity of various *Delftia* isolates and their ability to effectively suppress the growth of various plant pathogens. Furthermore, we have demonstrated its broad antibacterial activity against challenging MDR pathogens, alone or in synergy with other antibiotics, and the potential of utilizing it as a “Trojan-horse”.

## Data Availability Statement

The datasets generated for this study can be found in FLCF00000000.2 and ERR1314968.

## Author Contributions

NT-Y was responsible for experiments design, results analysis as well as manuscript writing. AR, YD, and AS were responsible for experiments design, performing, and analysis. AV was responsible for the HPLC method developments and delftibactin A purification. FR was responsible for NMR and LCMS/MS analysis of Delftibactin A. GR was responsible for results analysis as well as manuscript writing. IN was responsible for leading the research group, coordinating, experiments design, results analysis as well as manuscript writing.

## Conflict of Interest

The authors declare that the research was conducted in the absence of any commercial or financial relationships that could be construed as a potential conflict of interest.

## Footnotes

1 http://exploration.weizmann.ac.il/perl/pandatox1_0.mperl?submit=show_homologs&locus_tag=Daci_4752&replicon_accession=NC_010002
