# Supplementary material for: Delftibactin-A, a Non-ribosomal Peptide With Broad Antimicrobial Activity
Source: Front Microbiol. 2019 Oct 15;10:2377. doi: 10.3389/fmicb.2019.02377 (PMC6808179; doi:10.3389/fmicb.2019.02377)
Supplement: Supplementary file 1 [file Presentation_1.PPTX]

## Slide 1
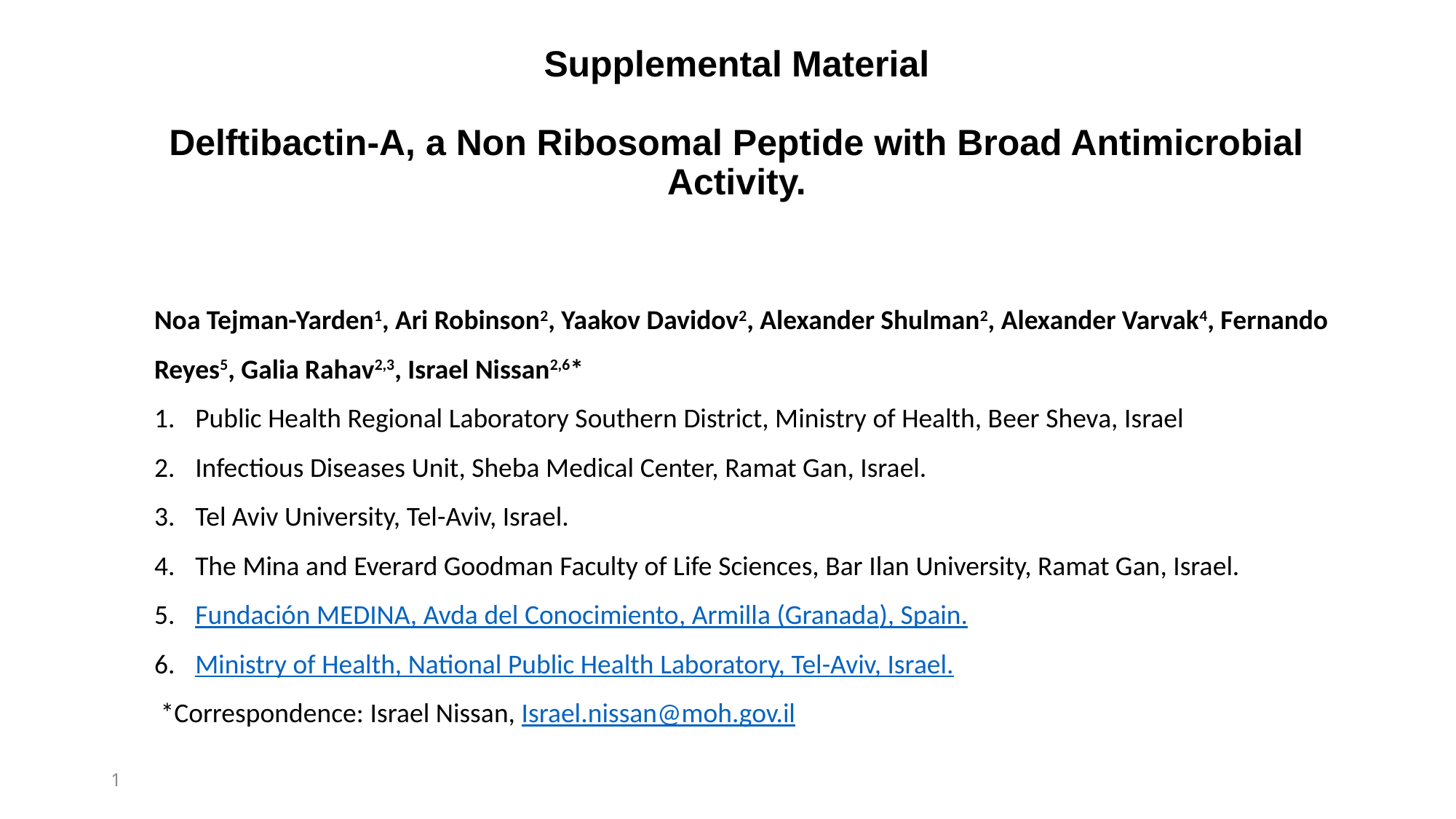

# Supplemental Material Delftibactin-A, a Non Ribosomal Peptide with Broad Antimicrobial Activity.
Noa Tejman-Yarden1, Ari Robinson2, Yaakov Davidov2, Alexander Shulman2, Alexander Varvak4, Fernando Reyes5, Galia Rahav2,3, Israel Nissan2,6*
Public Health Regional Laboratory Southern District, Ministry of Health, Beer Sheva, Israel
Infectious Diseases Unit, Sheba Medical Center, Ramat Gan, Israel.
Tel Aviv University, Tel-Aviv, Israel.
The Mina and Everard Goodman Faculty of Life Sciences, Bar Ilan University, Ramat Gan, Israel.
Fundación MEDINA, Avda del Conocimiento, Armilla (Granada), Spain.
Ministry of Health, National Public Health Laboratory, Tel-Aviv, Israel.
 *Correspondence: Israel Nissan, Israel.nissan@moh.gov.il
1

## Slide 2
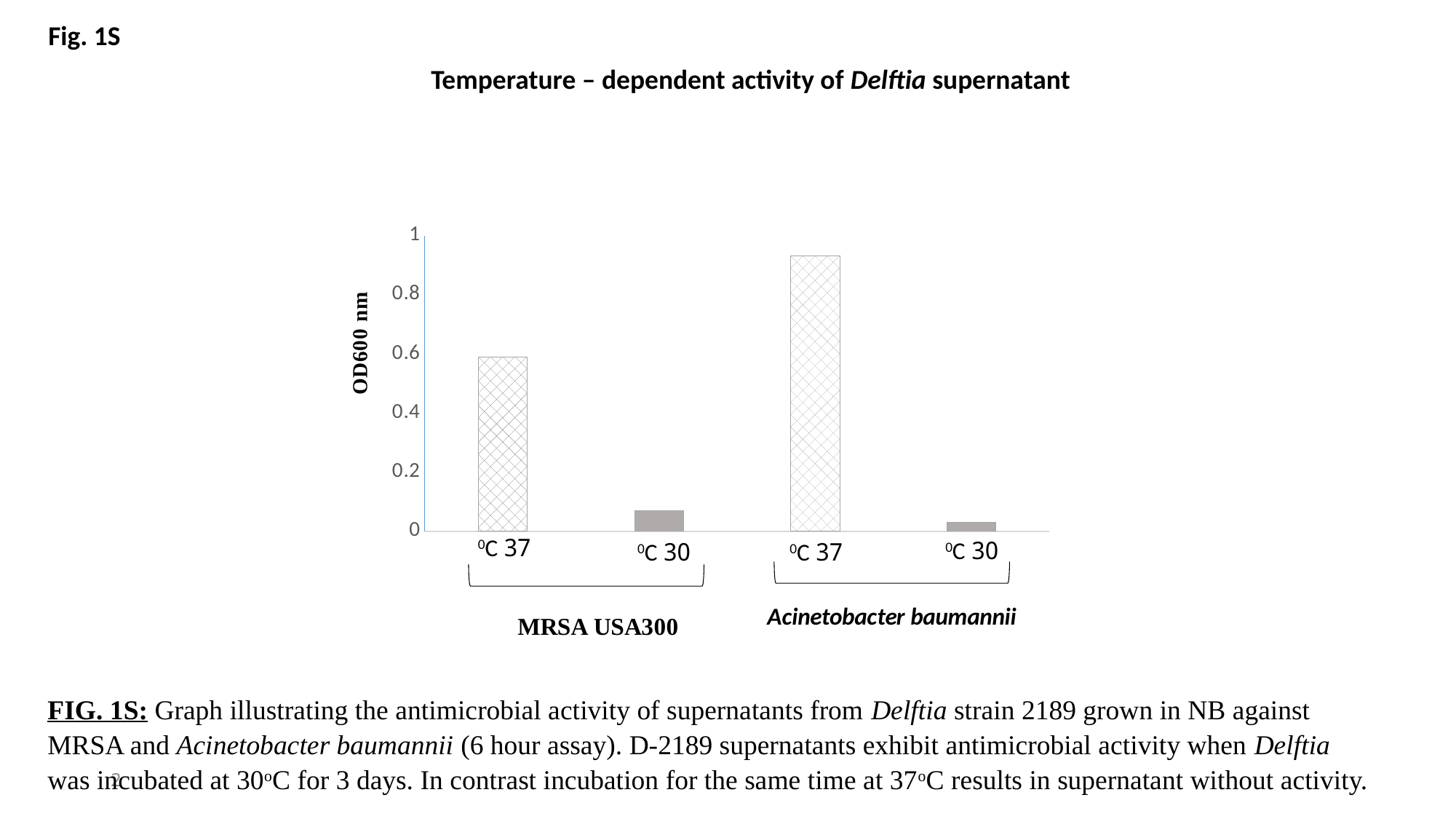

Fig. 1S
Temperature – dependent activity of Delftia supernatant
### Chart
| Category | |
|---|---|
| USA300 | 0.59 |
| USA300 | 0.07 |
| Acinetobacter | 0.93 |
| Acinetobacter | 0.03 |
37 0C
30 0C
30 0C
37 0C
FIG. 1S: Graph illustrating the antimicrobial activity of supernatants from Delftia strain 2189 grown in NB against MRSA and Acinetobacter baumannii (6 hour assay). D-2189 supernatants exhibit antimicrobial activity when Delftia was incubated at 30oC for 3 days. In contrast incubation for the same time at 37oC results in supernatant without activity.
2

## Slide 3
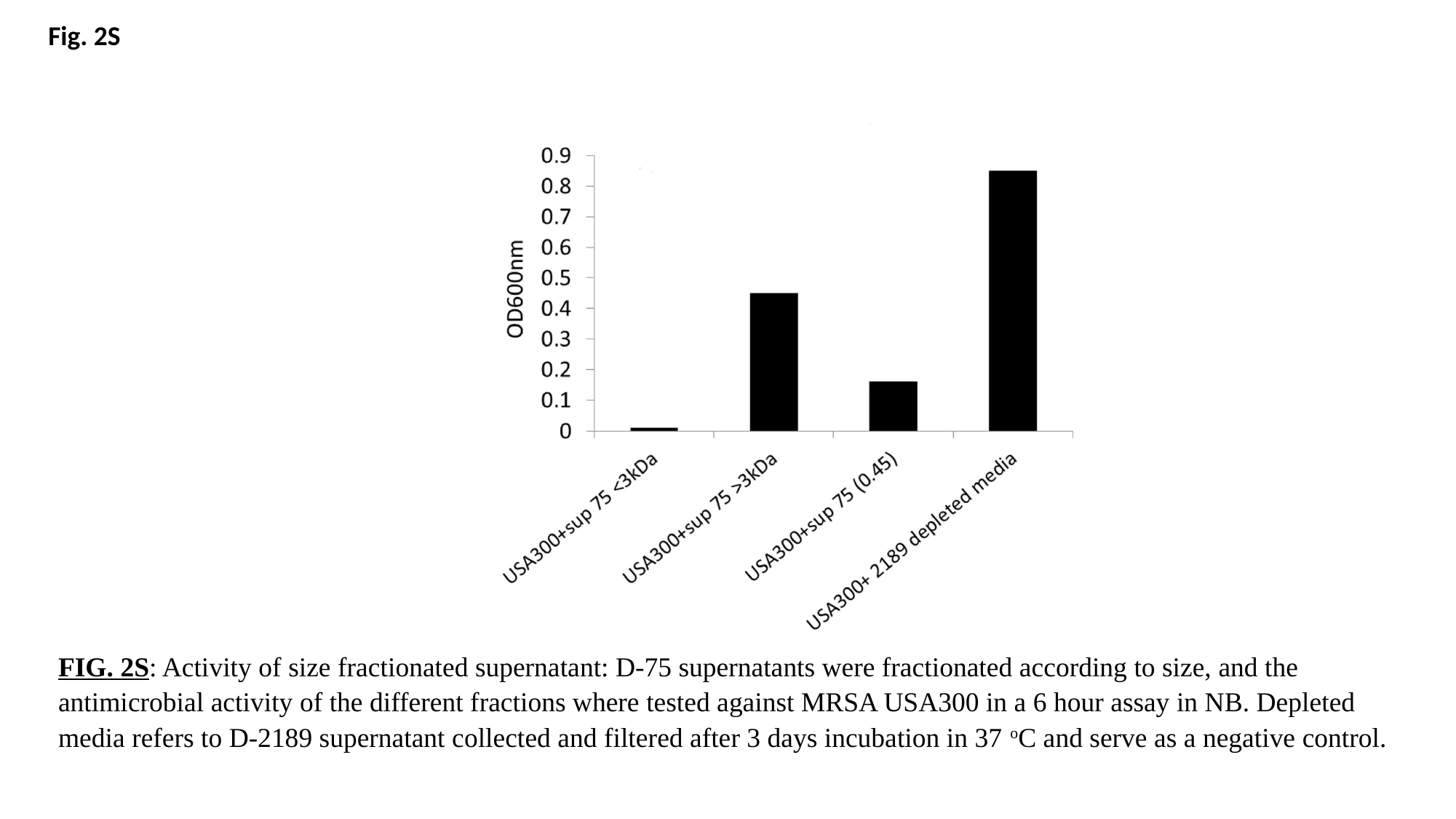

Fig. 2S
#
FIG. 2S: Activity of size fractionated supernatant: D-75 supernatants were fractionated according to size, and the antimicrobial activity of the different fractions where tested against MRSA USA300 in a 6 hour assay in NB. Depleted media refers to D-2189 supernatant collected and filtered after 3 days incubation in 37 oC and serve as a negative control.
3

## Slide 4
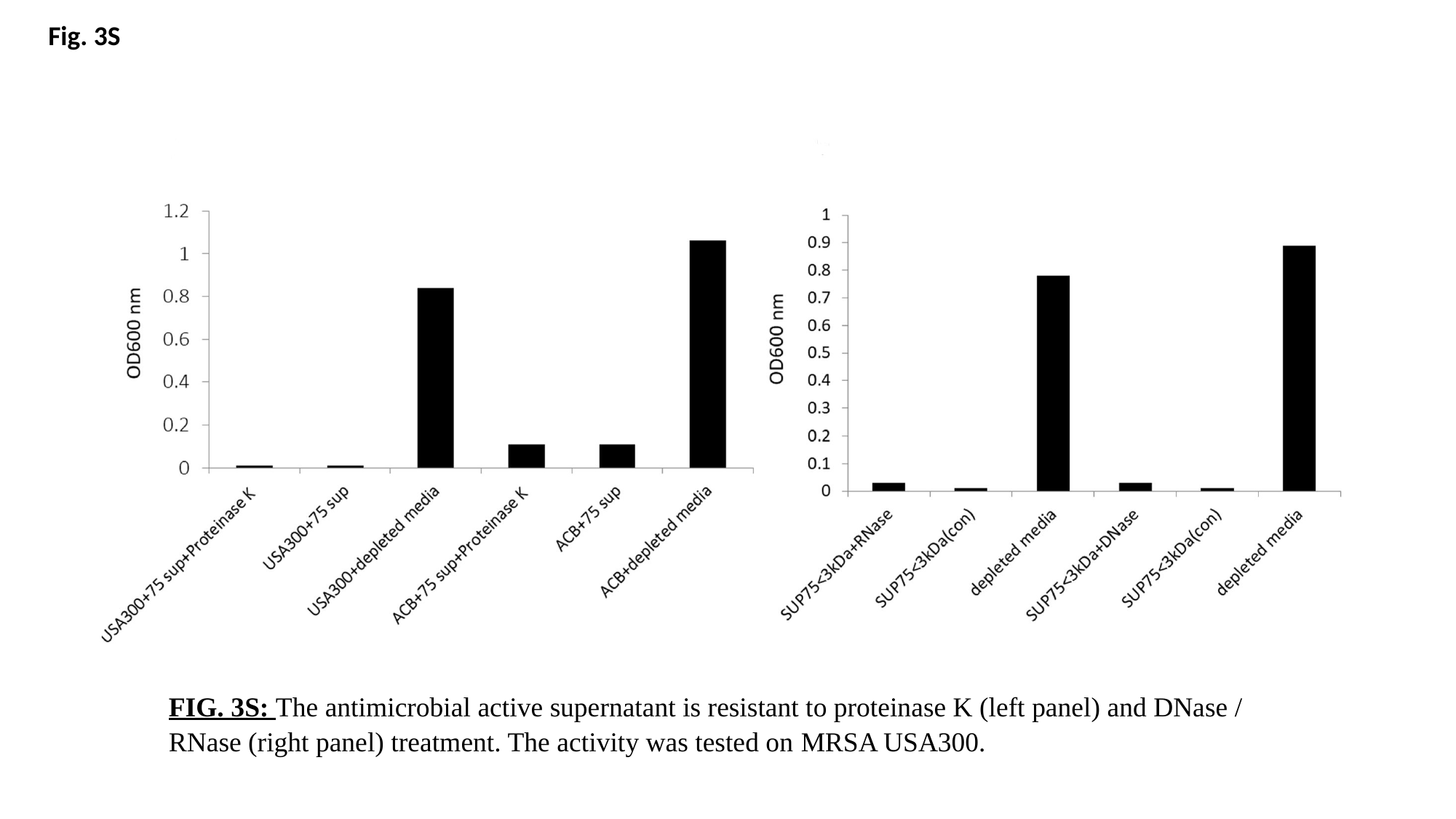

Fig. 3S
#
FIG. 3S: The antimicrobial active supernatant is resistant to proteinase K (left panel) and DNase / RNase (right panel) treatment. The activity was tested on MRSA USA300.
4

## Slide 5
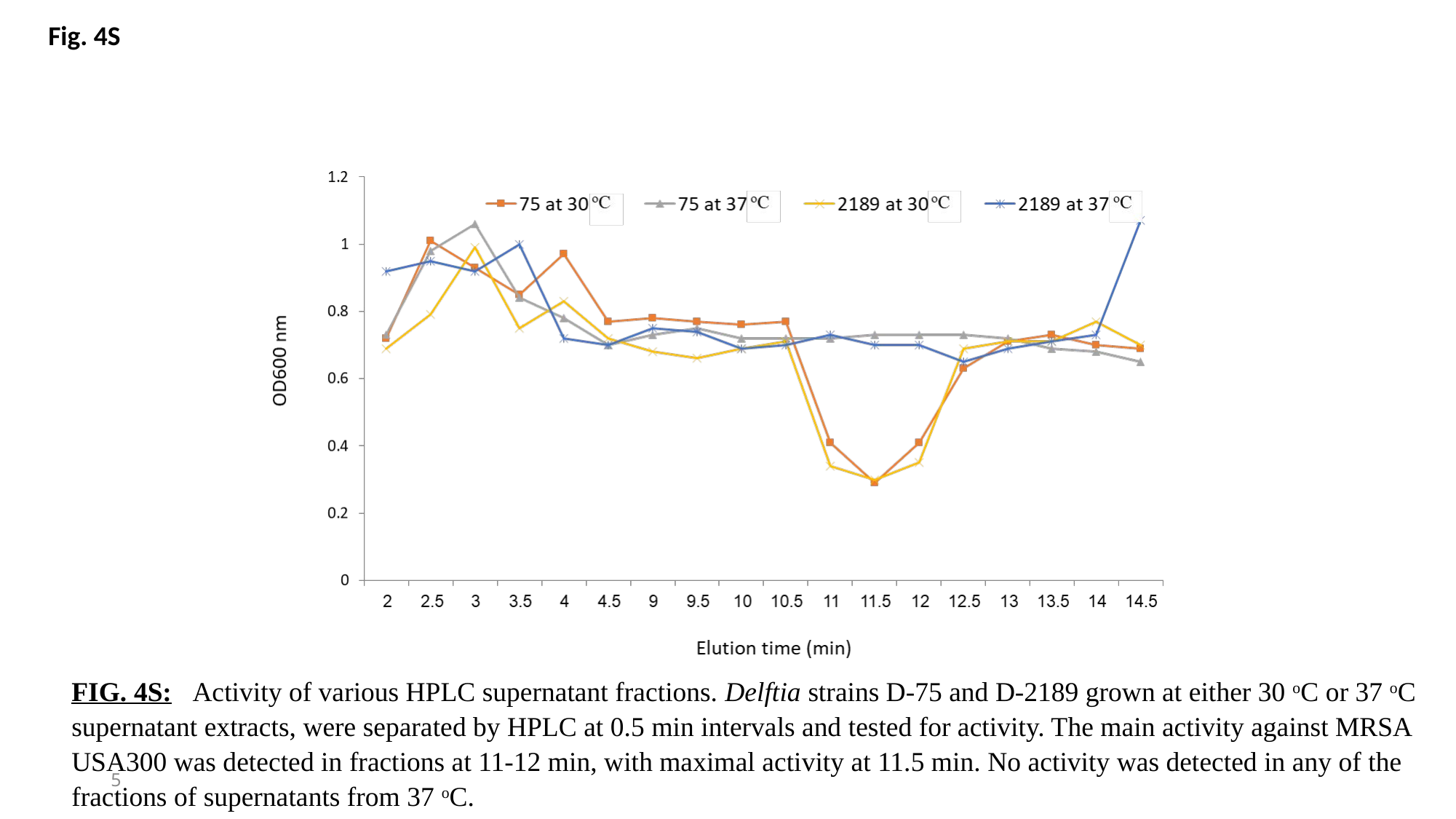

Fig. 4S
FIG. 4S: Activity of various HPLC supernatant fractions. Delftia strains D-75 and D-2189 grown at either 30 oC or 37 oC supernatant extracts, were separated by HPLC at 0.5 min intervals and tested for activity. The main activity against MRSA USA300 was detected in fractions at 11-12 min, with maximal activity at 11.5 min. No activity was detected in any of the fractions of supernatants from 37 oC.
5

## Slide 6
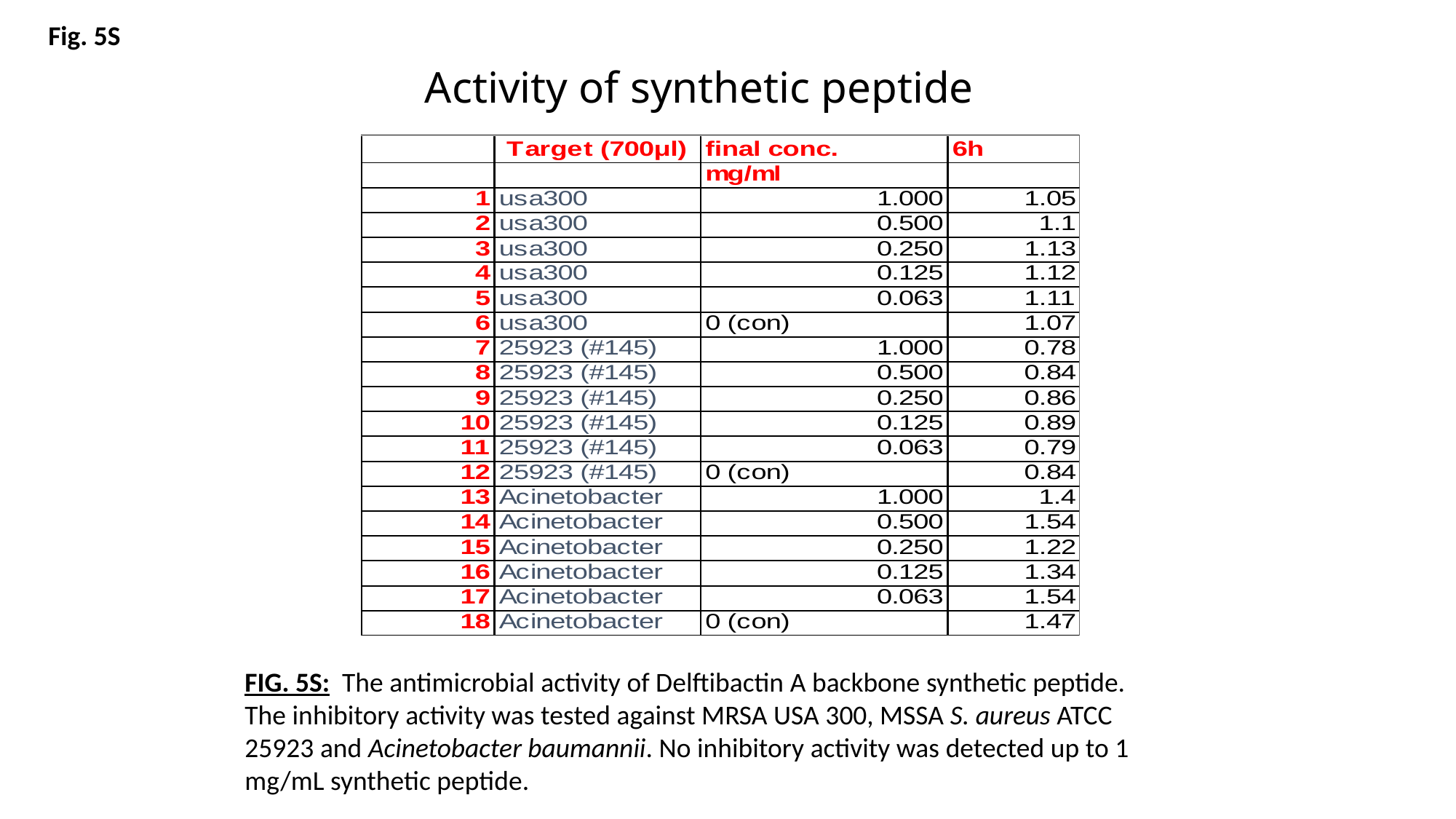

Fig. 5S
# Activity of synthetic peptide
FIG. 5S: The antimicrobial activity of Delftibactin A backbone synthetic peptide. The inhibitory activity was tested against MRSA USA 300, MSSA S. aureus ATCC 25923 and Acinetobacter baumannii. No inhibitory activity was detected up to 1 mg/mL synthetic peptide.

## Slide 7
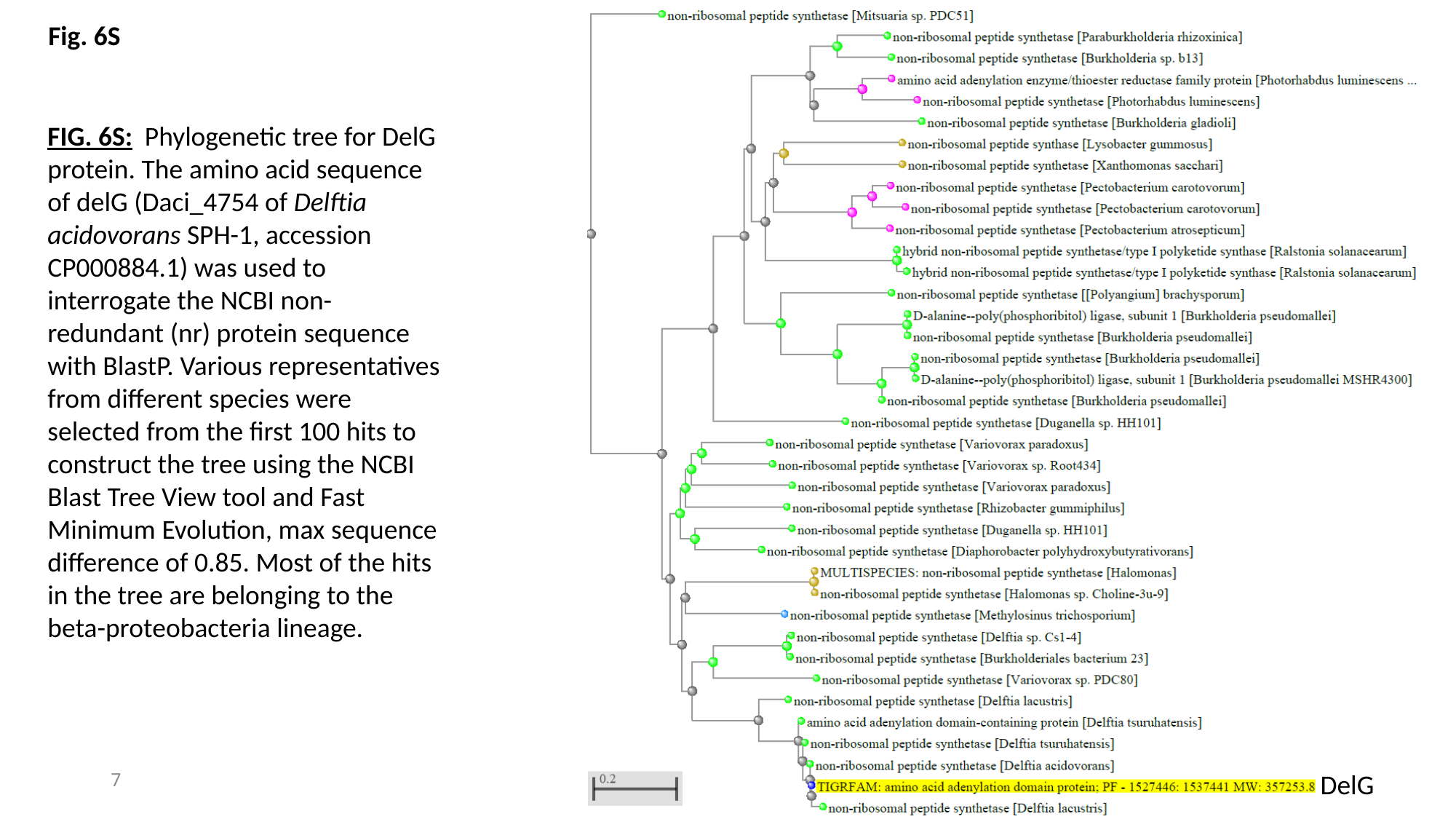

Fig. 6S
FIG. 6S: Phylogenetic tree for DelG protein. The amino acid sequence of delG (Daci_4754 of Delftia acidovorans SPH-1, accession CP000884.1) was used to interrogate the NCBI non-redundant (nr) protein sequence with BlastP. Various representatives from different species were selected from the first 100 hits to construct the tree using the NCBI Blast Tree View tool and Fast Minimum Evolution, max sequence difference of 0.85. Most of the hits in the tree are belonging to the beta-proteobacteria lineage.
7
DelG

## Slide 8
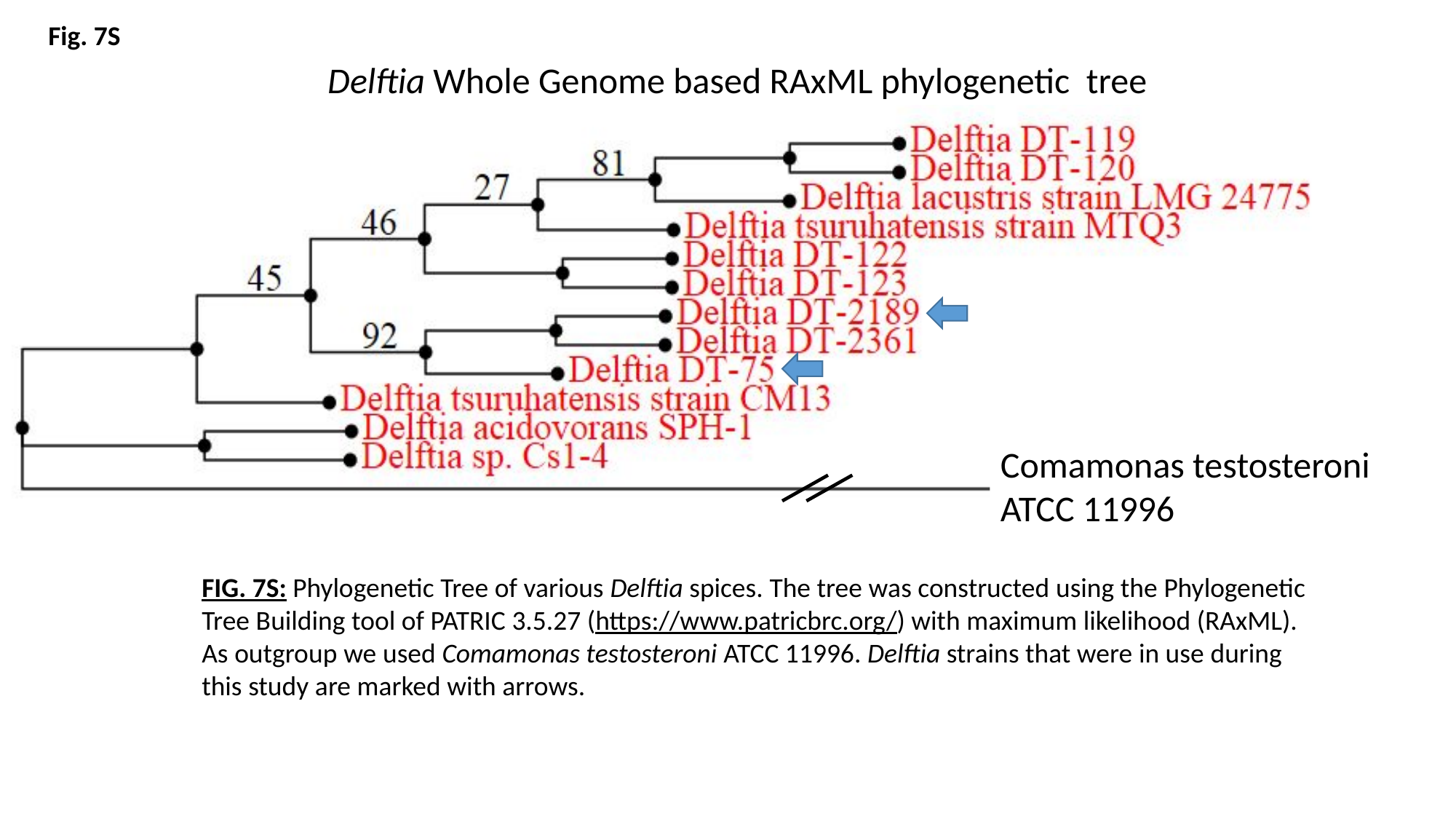

Fig. 7S
Delftia Whole Genome based RAxML phylogenetic tree
Comamonas testosteroni
ATCC 11996
FIG. 7S: Phylogenetic Tree of various Delftia spices. The tree was constructed using the Phylogenetic Tree Building tool of PATRIC 3.5.27 (https://www.patricbrc.org/) with maximum likelihood (RAxML). As outgroup we used Comamonas testosteroni ATCC 11996. Delftia strains that were in use during this study are marked with arrows.

## Slide 9
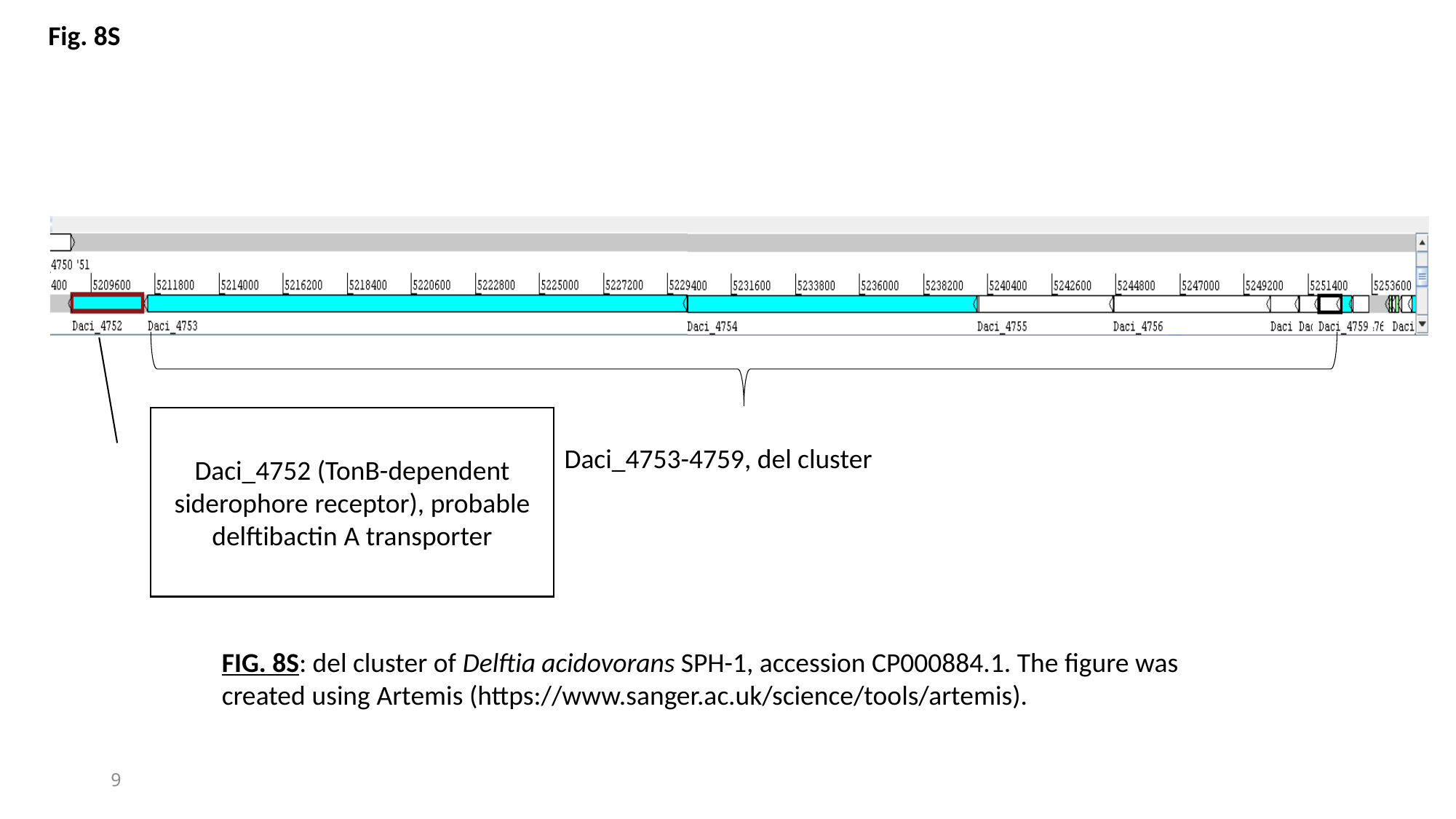

Fig. 8S
Daci_4752 (TonB-dependent siderophore receptor), probable delftibactin A transporter
Daci_4753-4759, del cluster
FIG. 8S: del cluster of Delftia acidovorans SPH-1, accession CP000884.1. The figure was created using Artemis (https://www.sanger.ac.uk/science/tools/artemis).
9

## Slide 10
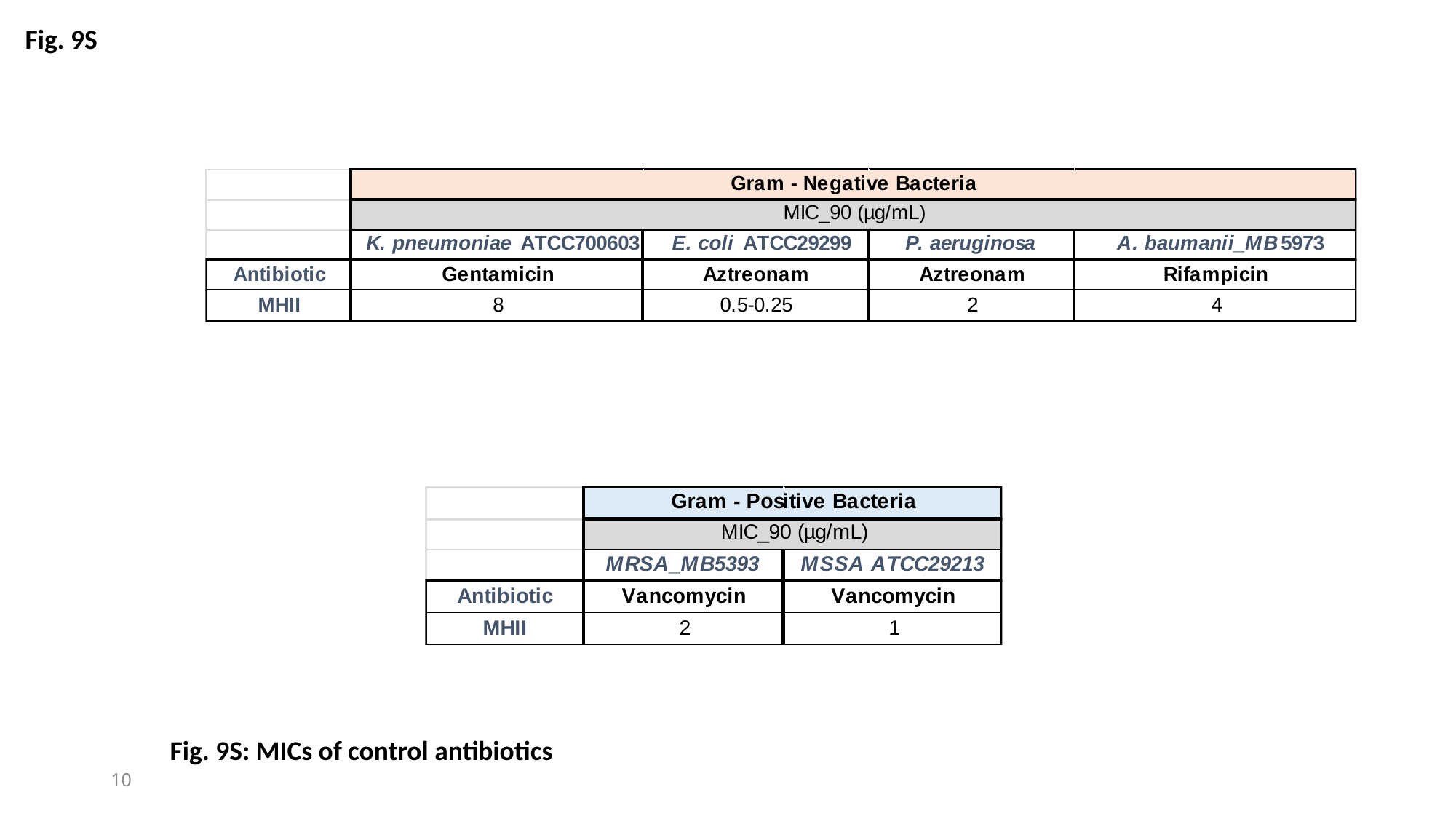

Fig. 9S
Fig. 9S: MICs of control antibiotics
10

## Slide 11
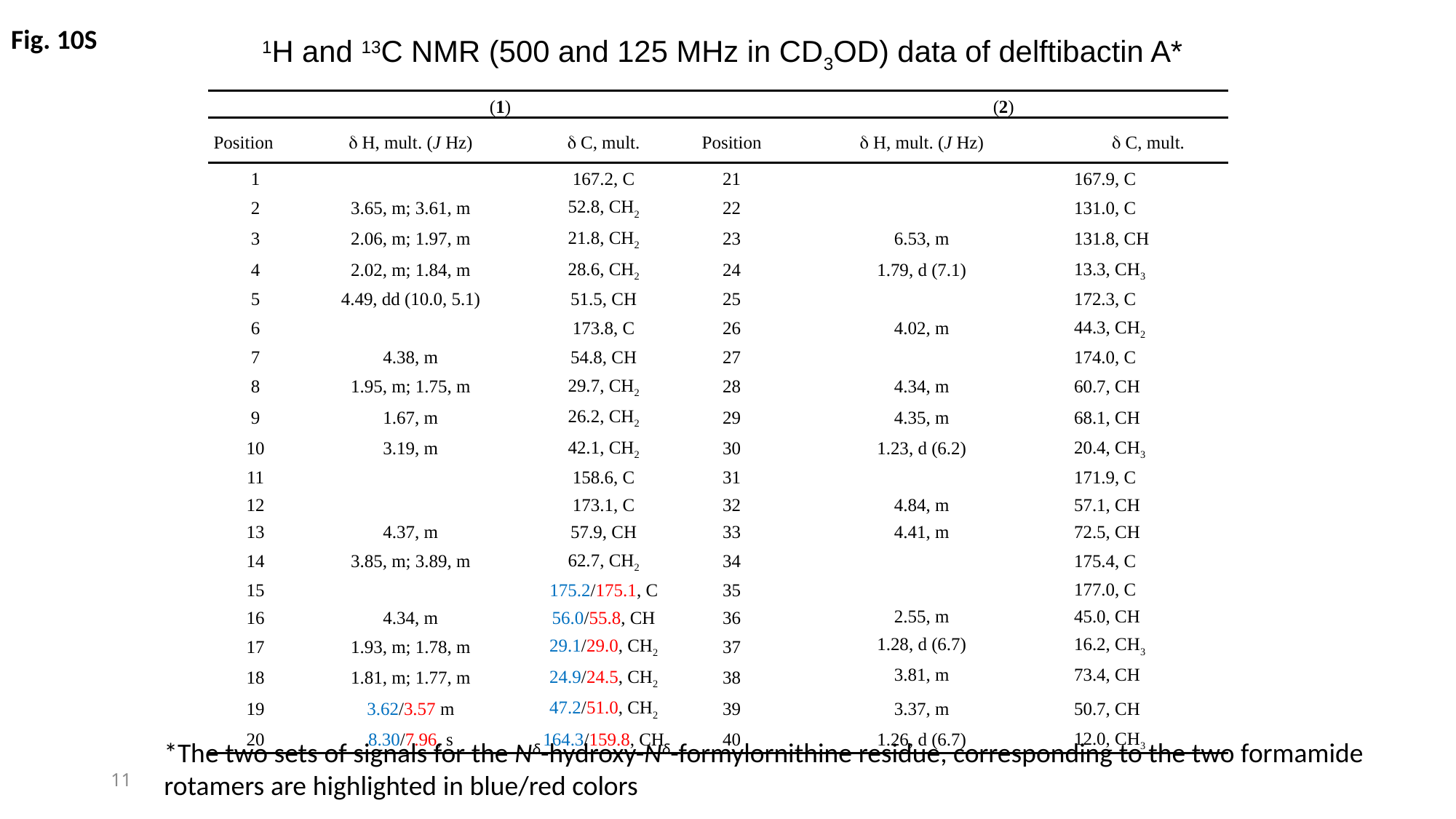

Fig. 10S
1H and 13C NMR (500 and 125 MHz in CD3OD) data of delftibactin A*
| | (1) | | | (2) | |
| --- | --- | --- | --- | --- | --- |
| Position | d H, mult. (J Hz) | d C, mult. | Position | d H, mult. (J Hz) | d C, mult. |
| 1 | | 167.2, C | 21 | | 167.9, C |
| 2 | 3.65, m; 3.61, m | 52.8, CH2 | 22 | | 131.0, C |
| 3 | 2.06, m; 1.97, m | 21.8, CH2 | 23 | 6.53, m | 131.8, CH |
| 4 | 2.02, m; 1.84, m | 28.6, CH2 | 24 | 1.79, d (7.1) | 13.3, CH3 |
| 5 | 4.49, dd (10.0, 5.1) | 51.5, CH | 25 | | 172.3, C |
| 6 | | 173.8, C | 26 | 4.02, m | 44.3, CH2 |
| 7 | 4.38, m | 54.8, CH | 27 | | 174.0, C |
| 8 | 1.95, m; 1.75, m | 29.7, CH2 | 28 | 4.34, m | 60.7, CH |
| 9 | 1.67, m | 26.2, CH2 | 29 | 4.35, m | 68.1, CH |
| 10 | 3.19, m | 42.1, CH2 | 30 | 1.23, d (6.2) | 20.4, CH3 |
| 11 | | 158.6, C | 31 | | 171.9, C |
| 12 | | 173.1, C | 32 | 4.84, m | 57.1, CH |
| 13 | 4.37, m | 57.9, CH | 33 | 4.41, m | 72.5, CH |
| 14 | 3.85, m; 3.89, m | 62.7, CH2 | 34 | | 175.4, C |
| 15 | | 175.2/175.1, C | 35 | | 177.0, C |
| 16 | 4.34, m | 56.0/55.8, CH | 36 | 2.55, m | 45.0, CH |
| 17 | 1.93, m; 1.78, m | 29.1/29.0, CH2 | 37 | 1.28, d (6.7) | 16.2, CH3 |
| 18 | 1.81, m; 1.77, m | 24.9/24.5, CH2 | 38 | 3.81, m | 73.4, CH |
| 19 | 3.62/3.57 m | 47.2/51.0, CH2 | 39 | 3.37, m | 50.7, CH |
| 20 | 8.30/7.96, s | 164.3/159.8, CH | 40 | 1.26, d (6.7) | 12.0, CH3 |
*The two sets of signals for the Nδ-hydroxy-Nδ-formylornithine residue, corresponding to the two formamide rotamers are highlighted in blue/red colors
11

## Slide 12
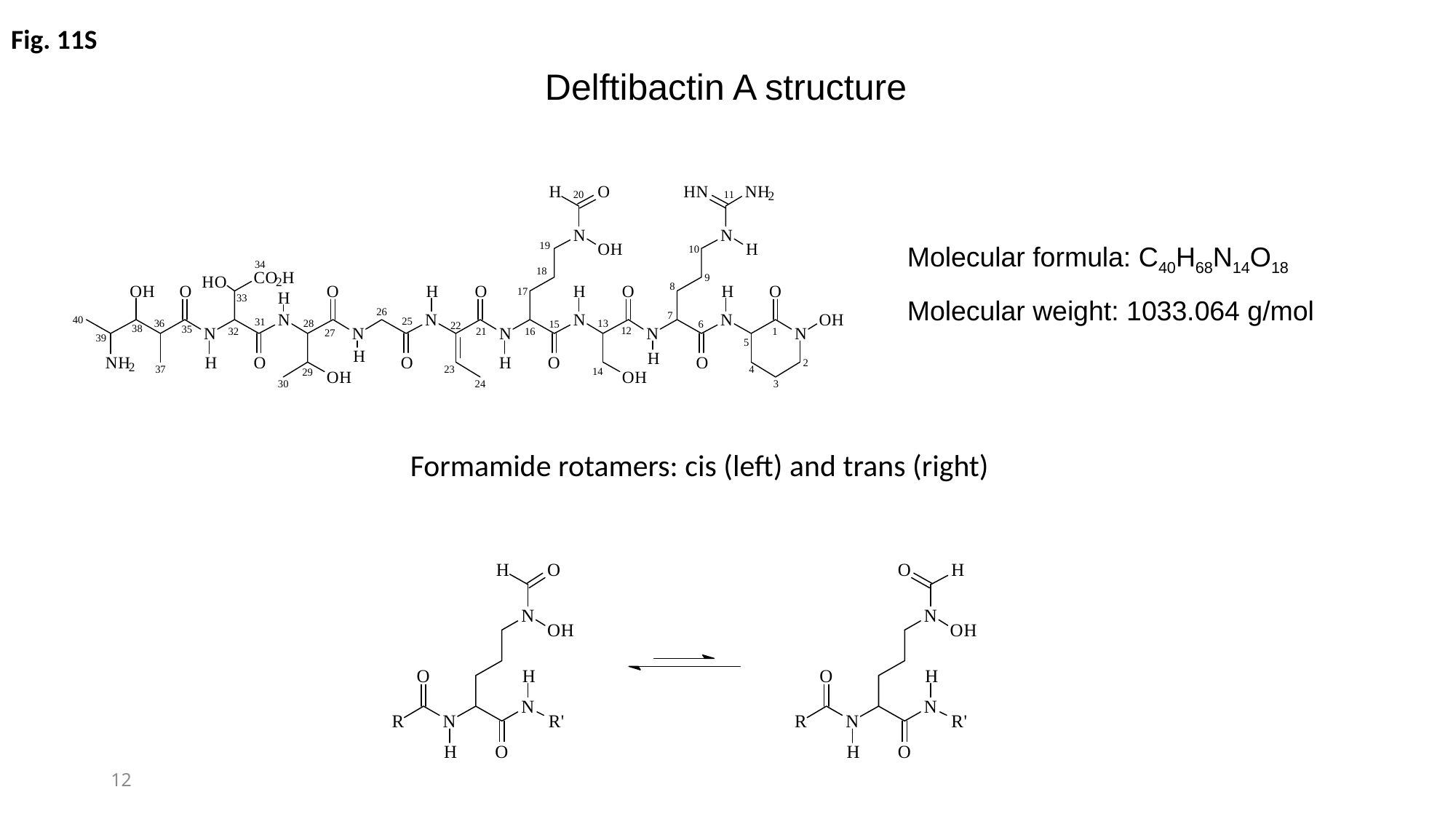

Fig. 11S
Delftibactin A structure
Molecular formula: C40H68N14O18
Molecular weight: 1033.064 g/mol
Formamide rotamers: cis (left) and trans (right)
12

## Slide 13
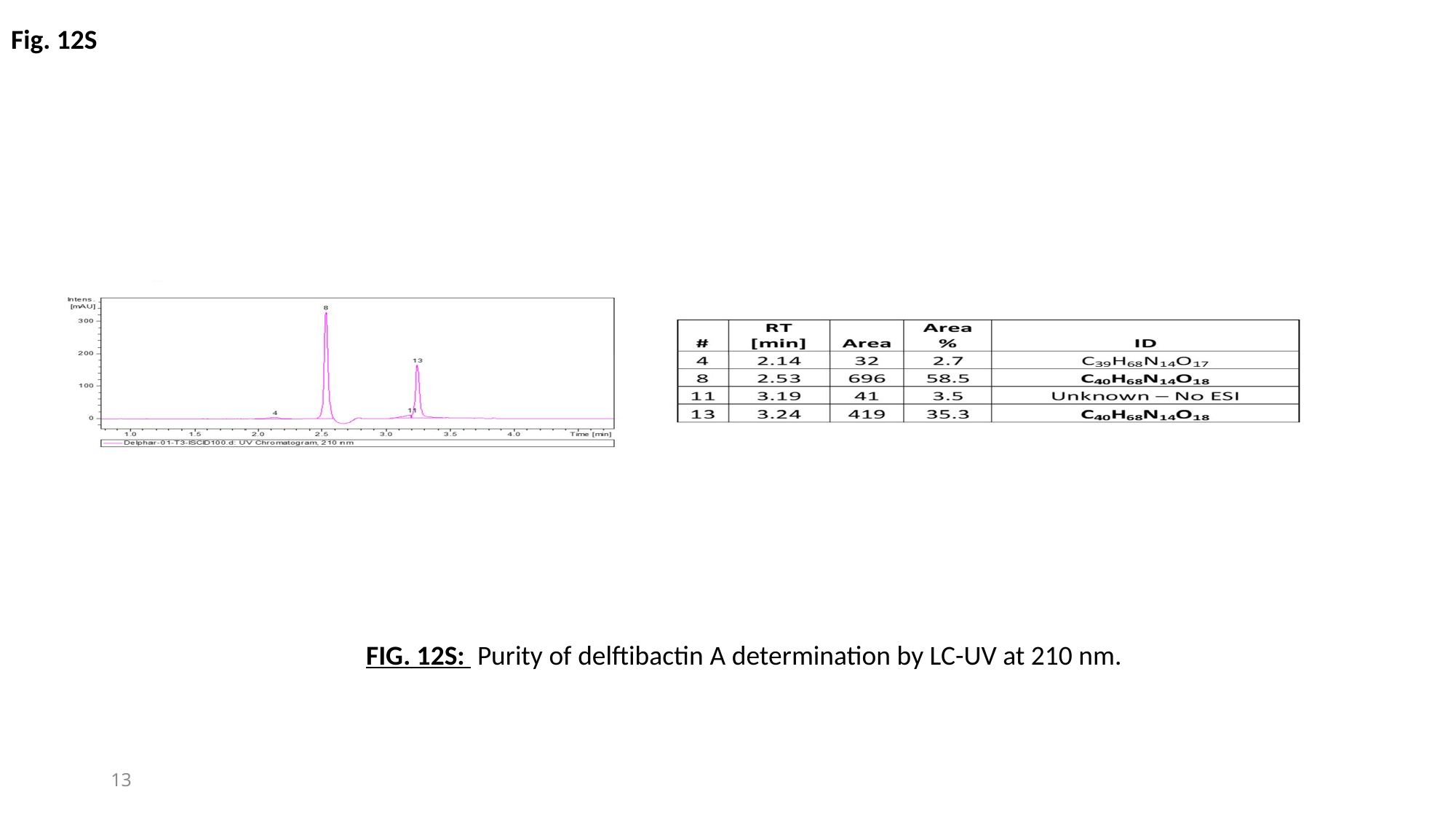

Fig. 12S
FIG. 12S: Purity of delftibactin A determination by LC-UV at 210 nm.
13

## Slide 14
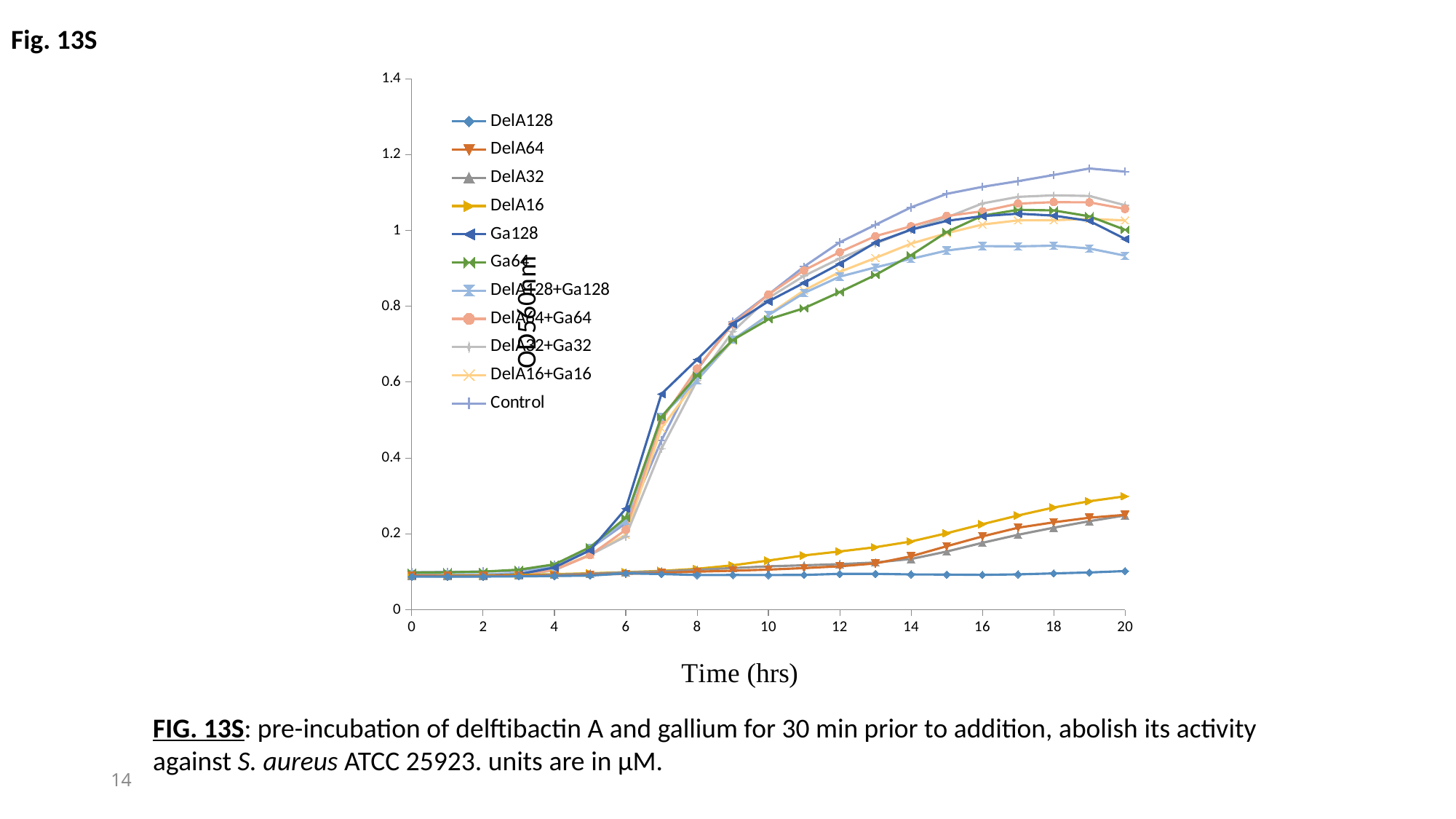

Fig. 13S
### Chart
| Category | DelA128 | DelA64 | DelA32 | DelA16 | Ga128 | Ga64 | DelA128+Ga128 | DelA64+Ga64 | DelA32+Ga32 | DelA16+Ga16 | Control |
|---|---|---|---|---|---|---|---|---|---|---|---|FIG. 13S: pre-incubation of delftibactin A and gallium for 30 min prior to addition, abolish its activity against S. aureus ATCC 25923. units are in µM.
14
